# Laboratory-Assessed Markers of Cardiometabolic Health and Associations with GIS-Based Measures of Active-Living Environments

**DOI:** 10.3390/ijerph15102079

**Published:** 2018-09-21

**Authors:** Samantha Hajna, Kaberi Dasgupta, Nancy A. Ross

**Affiliations:** 1 MRC Epidemiology Unit, Institute of Metabolic Science, University of Cambridge School of Clinical Medicine, Cambridge Biomedical Campus, Cambridge CB2 0QQ, UK; samantha.hajna@mrc-epid.cam.ac.uk; 2 Department of Geography, McGill University, 805 Sherbrooke St. West, Montréal, QC H3A 0B9, Canada; 3 Department of Epidemiology, Biostatistics and Occupational Health, McGill University, 1020 Pine Ave. West, Montréal, QC H3A 1A2, Canada; kaberi.dasgupta@mcgill.ca; 4 Department of Medicine, Divisions of Internal Medicine, Clinical Epidemiology, and Endocrinology and Metabolism, McGill University Health Centre, Montréal, QC H4A 3J1, Canada

**Keywords:** neighborhood physical-activity environments, cardiometabolic health, body-mass index, blood pressure, physical activity

## Abstract

Active-living-friendly environments have been linked to physical activity, but their relationships with specific markers of cardiometabolic health remain unclear. We estimated the associations between active-living environments and markers of cardiometabolic health, and explored the potential mediating role of physical activity in these associations. We used data collected on 2809 middle-aged adults who participated in the Canadian Health Measures Survey (2007–2009; 41.5 years, SD = 15.1). Environments were assessed using an index that combined GIS-derived measures of street connectivity, land use mix, and population density. Body mass index (BMI), systolic blood pressure (SBP), hemoglobin A1c, and cholesterol were assessed in a laboratory setting. Daily step counts and moderate-to-vigorous intensity physical activity (MVPA) were assessed for seven days using accelerometers. Associations were estimated using robust multivariable linear regressions adjusted for sociodemographic factors that were assessed via questionnaire. BMI was 0.79 kg/m^2^ lower (95% confidence interval (CI) −1.31, −0.27) and SBP was 1.65 mmHg lower (95% CI −3.10, −0.20) in participants living in the most active-living-friendly environments compared to the least, independent of daily step counts or MVPA. A 35.4 min/week difference in MPVA (95% CI 24.2, 46.6) was observed between residents of neighborhoods in the highest compared to the lowest active-living-environment quartiles. Cycling to work rates were also the highest in participants living in the highest living-environment quartiles (e.g., Q4 vs. Q1: 10.4% vs. 4.9%). Although active-living environments are associated with lower BMI and SBP, and higher MVPA and cycling rates, neither daily step counts nor MVPA appear to account for environment–BMI/SBP relationships. This suggests that other factors not assessed in this study (e.g., food environment or unmeasured features of the social environment) may explain this relationship.

## 1. Introduction

Lower rates of obesity [1,2,3,4], type 2 diabetes [1,2,3,5,6], insulin resistance [7], and hypertension [4,8] have been observed among adults living in neighborhoods with more connected street networks, a greater intermixing of land uses, and higher population densities. While these studies have advanced our understanding of the importance of environments on human health, population-based studies that link neighborhood active-living environments to a wider range of laboratory-assessed markers of cardiometabolic health, and that also consider the potential mediating role of physical activity in these relationships are lacking. Most people spend considerable periods of time in their home neighborhoods. As a result, their health behaviors (e.g., physical-activity choices) might be directly influenced by how supportive their neighborhoods are for these types of behaviors. In turn, these environments may have important implications for physical activity-related health outcomes, such as cardiometabolic disease risk. Indeed, higher physical-activity levels are associated with more favorable cardiometabolic health profiles [9,10]. Several studies have, however, failed to find a link between objectively assessed environments and step counts [11,12,13]. It is possible that higher-intensity physical activity might be the mediating factor; alternatively, other neighborhood factors (e.g., food environments) might be important. The aim of our study was to estimate the associations between neighborhood active-living-friendliness (based on street connectivity, land use mix, population density) and several markers of cardiometabolic health. Our secondary aim was to explore the potential mediating roles of daily step counts and moderate-to-vigorous intensity physical activity (MVPA) in these relationships.

## 2. Materials and Methods

### 2.1. Study Population

We used data collected between March 2007 and February 2009 on middle-aged adults who participated in the Canadian Health Measures Survey (CHMS; Cycle 1). The CHMS is a biennial population-based study of a representative sample of Canadians [14]. Data collection involved a computer-assisted questionnaire and a mobile-clinic assessment at 15 sites located in one of 5 geographic regions across Canada. The number of sites per geographic region was proportional to the size of the population. Individuals living in Indigenous communities or in long-term care institutions and those who were full-time members of the Canadian Forces were not eligible to participate. The CHMS was approved by the Health Canada Research Ethics Board (REB#2005-0025). Statistics Canada obtained written informed consent from all participants prior to data collection. We retained participants aged ≥18 years and excluded participants who were taking glucose-lowering, antihypertensive, and/or cholesterol-lowering medications. Data access was granted by the Social Sciences and Humanities Research Council of Canada (12-SSH-MCG-3081). Analyses were performed at the McGill-Concordia Quebec Inter-University Center for Social Statistics (QICSS).

### 2.2. Neighborhood Active-Living Environments

Neighborhoods were defined as 500 m polygonal buffers around the centroid of the participants’ six-digit home postal codes [12]. Postal codes are very accurate proxies for home addresses in the Canadian context: 87.9% and 96.5% of Canadian postal codes fall within 200 and 500 m of the true home address, respectively [15]. Further to this, when converting six-digit postal codes to latitudes/longitudes, we used Statistic Canada’s Postal Code Conversion File Plus (PCCF+). This file allowed us to use population-weighted random allocation to increase the probability of assigning a latitude/longitude that would be in close proximity to the residentially zoned areas and thereby minimized the possibility that the derived buffers would not include the participants’ actual addresses [15]. Five-hundred-meter buffers were selected as they have been assumed to approximate a 10 min walking distance [16]. Street connectivity, land use mix, and population density were calculated for each buffer using ArcMap 10.1 (ESRI; Redlands, CA, USA). Street connectivity was calculated as the number of ≥3-way intersections/km^2^ in each neighborhood. Land use mix represented the degree of heterogeneity in residential, commercial, institutional/governmental, and recreational land uses and was calculated using the formula: land use mix = (−1) Σ_k_(P_k_lnP_k_)/ln N, where P represented the proportion of land area devoted to the specific land use (k) in each buffer divided by ln (4) [17,18]. The land use mix score ranged from 0 to 1. A higher value indicated greater diversity in land uses contained within the home neighborhood. Population density represented the unadjusted census population counts/km^2^ in the dissemination area that corresponded to each postal code. We calculated street connectivity and land use mix using 2009 DMTI CanMap^®^ Streetfiles (DMTI Spatial Inc., Markham, ON, Canada) [19], and population density using the 2006 Canadian Census Population Counts File [20]. The z-scores of street connectivity, population density, and land use mix were summed to create a neighborhood active-living environment index. A higher index represented neighborhoods with more potential to be active-living-friendly by having more connected street networks, greater intermixing of land uses, and more people. Areas that are very active-living-friendly tend to be located in the downtown areas of the largest Canadian cities, while older suburban, new suburban, and rural locations, tend to, in turn, have lower index scores.

### 2.3. Markers of Cardiometabolic Health

Body mass index (BMI), systolic blood pressure (SBP), hemoglobin A1c, and lipid profiles, well-established predictors of cardiovascular outcomes [21,22,23,24,25,26], were assessed as part of the mobile-clinic evaluation. Height and weight were assessed using the Canadian Physical Activity, Fitness, and Lifestyle Approach (CPAFLA) protocol [27] and used to calculated BMI (kg/m^2^). Specifically, weight was assessed using a digital scale (VLC^TM^, Mettler Toledo, Columbus, OH, USA), and standing height was assessed using a fixed stadiometer (ProScale M150; Accurate Technology Inc., Fletcher, NC, USA). SBP (mmHg) was assessed using an automated oscillometer (BpTRU^TM^ BP-300, BpTRU Medical Devices, Coquitlam, BC, Canada) after 5 min of rest. Six consecutive blood pressure measurements were taken, and the last 5 were used in the calculation of average SBP [28]. Hemoglobin A1c (%), and the total cholesterol/HDL cholesterol ratio were derived from blood samples collected by a phlebotomist using a standardized protocol, and analyzed in 1 of the 3 CHMS reference laboratories using standardized instrumentation (Vitros 5.1 FS, Ortho Clinical Diagnostics, Markham, ON, Canada).

### 2.4. Physical Activity

Participants wore a triaxial accelerometer (Actical; Philips-Respironics, Bend, OR, USA) on the right hip for 7 days following the mobile-clinic evaluation, except when bathing. The monitors were set to begin data collection at midnight after the clinic evaluation. Participants returned their units to Statistics Canada after the 7 day monitoring period using a postage-paid envelope [29]. The data were processed by Statistics Canada using previously published guidelines [30,31]. In brief, only participants with ≥4 valid days of data, with ≥10 h of data on each day, were retained in the analyses [14,30]. Steps accumulated over the monitoring period were divided by the number of days in which valid steps were registered for an estimate of daily step counts. MVPA was defined as any activity ≥1535 counts per minute. The step count and MVPA functions of Actical have been validated in adults [32].

### 2.5. Covariates

Age, sex, married/common-law status, education (having a Bachelor degree or higher), having ever smoked, depressed mood (depression, bipolar disorder, mania, or dysthymia), having children at home aged <15 years, being employed outside of the home, annual household income (cut-off: ≥$40,000), immigrant status, and cycling to work were assessed using a computer-assisted questionnaire.

### 2.6. Statistical Analyses

Descriptive statistics were produced for all variables of interest. Robust linear regressions (m estimation with bisquare weighting) were used to estimate mean differences in each of the markers of cardiometabolic health across quartiles of the neighborhood active-living index. Models were unadjusted and adjusted for variables specified a priori as potential covariates or confounders. These included age, sex, married/common-law, education, smoking status, depressed mood, having children less than 15 years of age in home, working status, and BMI, as well as total PA and MVPA, as appropriate. Final models were based on complete case data. All analyses were conducted in 2016 using SAS 9.4 (SAS Institute Inc., Cary, NC, USA).

## 3. Results

### 3.1. Participant Characteristics

A total of 3726 adults participated in Cycle 1 of the CHMS. After excluding respondents taking glucose-lowering medications (*n* = 192), antihypertensive medications (*n* = 772), and cholesterol-lowering medications (*n* = 407), data on 2809 adults were available. Participants averaged 41.5 years of age (SD = 15.1), there were more women than men (54.2% versus 45.8%), and most participants were married/common-law (58.3%) and employed (75.9%) (Table 1). Participants retained for analysis were younger (41.5 vs. 62.9 years), more educated (26.9% vs. 17.1% had a Bachelor’s degree or higher), and had better cardiometabolic profiles (e.g., SBP: 110.3 vs. 122.9 mmHg) than those who were excluded (Supplementary Table S1).

### 3.2. Neighborhood Active-Living Environments and Markers of Cardiometablic Health

**Figure 1:**
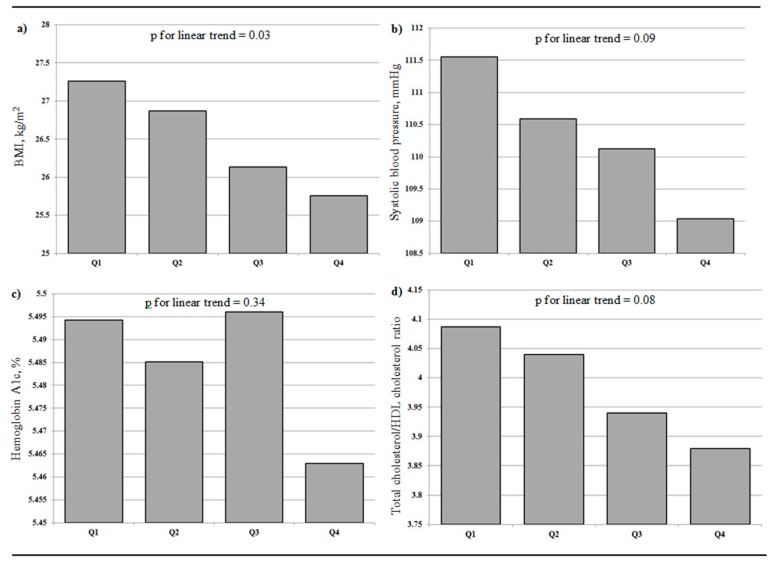
Mean levels of the cardiometabolic health markers of interest across quartiles of an index of neighborhood active-living friendliness (Q1: lowest neighborhood active-living index quartile; Q4: highest neighborhood active-living index quartile). (**a**) BMI; (**b**) systolic blood pressure; (**c**) hemoglobin A1c; (**d**) total cholesterol/HDL cholesterol ratio. Participants from the Canadian Health Measures Survey (2007–2009).

There was a graded inverse association between neighborhood active-living friendliness and several of the markers of cardiometabolic health. Participants living in more active-living-friendly neighborhoods had more optimal measures of BMI, SBP, hemoglobin A1c, and cholesterol (Figure 1; Table 2). BMI was 0.79 kg/m^2^ lower (95% confidence interval (CI) −1.31, −0.27) and SBP was 1.65 mmHg lower (95% CI −3.10, −0.20) in participants living in the highest compared to the lowest active-living quartiles, independent of sociodemographic characteristics and MVPA. Similar differences were observed when adjusting for total daily step counts instead of MVPA (Table 2).

### 3.3. Neighborhood Active-Living Environments, Daily Step Count, and MVPA

**Figure 2:**
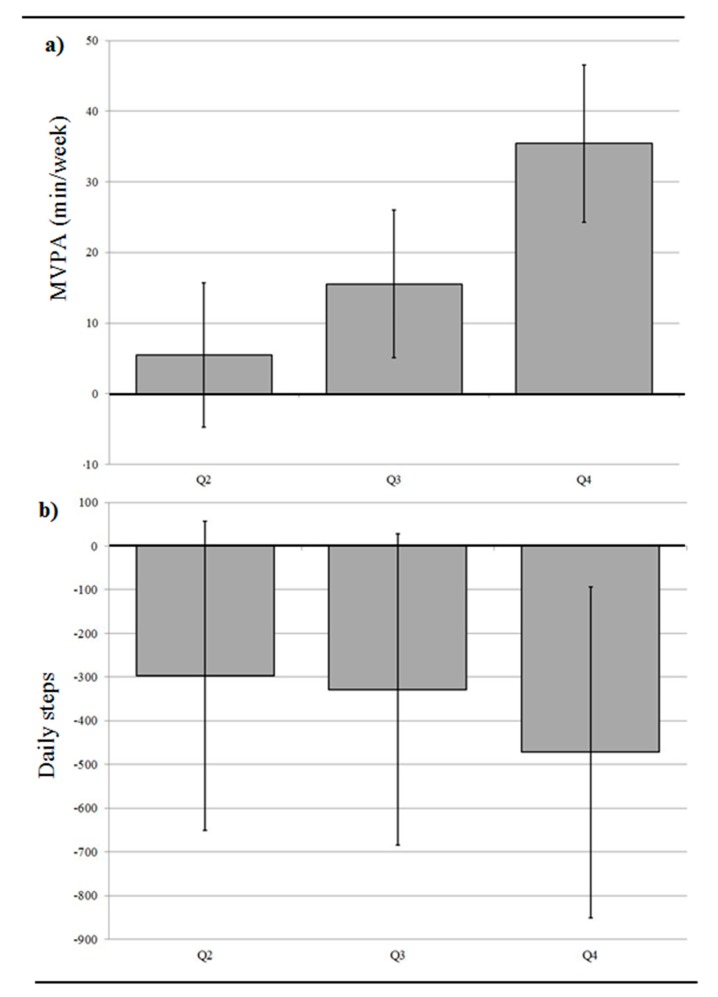
Differences in time spent in moderate-to-vigorous intensity physical activity (MVPA) and total accumulated daily steps (95% confidence intervals) in higher neighborhood active-living-friendliness index quartiles (Quartile 2, 3, and 4) compared to the lowest neighborhood active-living-friendliness index quartile (Quartile 1). (**a**) Differences in MVPA (min/week); (**b**) Differences in daily steps. Participants from the Canadian Health Measures Survey (2007–2009) **(**Models adjusted for age, sex, BMI, married/common-law status, children <15 years in household, annual household income, immigrant status, and depressed mood).

Participants living in more active-living-friendly neighborhoods accumulated fewer daily steps, but more MVPA, than participants living in less active-living-friendly neighborhoods (Figure 2). For example, a 35.4 min/week difference in MPVA (95% CI 24.2, 46.6) was observed between residents of neighborhoods in the highest compared to the lowest active-living environment quartiles. Differences in MVPA were smaller in the second and third quartiles, but still notable: Q3 vs. Q1: 15.5 min/week more MVPA (95% CI 5.1, 26.0) and Q2 vs. Q1: 5.5 min/week more MVPA (95% CI −4.8, 15.7). There was a moderate correlation between daily step counts and min/week in MVPA (R = 0.62; *n* = 2251). The percentage of people reporting cycling to work was also graded by neighborhood quartile: 10.4%, 7.9%, 6.7%, and 4.9% in the highest through to the lowest neighborhood quartiles, respectively.

## 4. Discussion

We found more favorable BMI and SBP values (i.e., 0.79 kg/m^2^ lower BMI; 1.65 mmHg lower SBP) in residents of the most compared to the least active-living-friendly neighborhoods. These associations were independent of sociodemographic characteristics, daily step counts, and MVPA. Our findings suggest that, while neighborhood features may support active living, there are other factors that likely explain the observed environment–cardiometabolic relationships. We found that participants living in the most compared to the least active-living-friendly neighborhoods accumulated 35 min/week more MVPA (they also had higher cycling-to-work rates), but the increased MVPA did not account for the favorable cardiometabolic profiles measured in these residents.

Our findings are in line with large-scale Canadian [2] and Swedish [6] studies of neighborhood active-living environments and type 2 diabetes, which suggest that this relationship is influenced by BMI (i.e., body weight is lower in more active-living-friendly environments). Our findings are also consistent with our previous longitudinal study showing that exposure to well-connected, mixed, and/or densely-populated neighborhoods is associated with reductions in BMI that are independent of socioeconomic characteristics and self-reported physical activity [33]. Given that socioeconomic and individual factors do not account for the favorable cardiometabolic profiles of residents of active-living-friendly environments, interventions aimed at creating more connected, mixed, and/or densely populated neighborhoods have the potential to reduce social inequalities in cardiometabolic health.

We did not find a neighborhood environment–hemoglobin A1c or –cholesterol association in this study. These results may be an artefact of restricting the sample to those not taking glucose-lowering—and cholesterol-lowering medications. That said, the largest group excluded from our analyses was of participants on antihypertensive medications, and we did identify an association between active-living environments and lower SBP, in line with evidence from other studies [4]. From a prevention perspective, excluding those on glucose-lowering, antihypertensive, and cholesterol-lowering medications remains important for the interpretation of the true relationship between the environment and markers of cardiometabolic health.

Adjusting for sociodemographic factors, daily step counts, and MVPA did not eliminate associations with BMI and SBP, suggesting that there are other neighborhood factors that are driving better cardiometabolic health. It is plausible that the neighborhood retail-food environment is a factor that may partly account for the associations others and we have observed with BMI and SBP. In a Canadian study of 7079 adults, younger adults who lived in a neighborhood with the greatest compared to the lowest proportion of fast-food outlets had a 79% (95% CI 1.03, 3.12) increased risk of developing diabetes over five years of follow-up [34]. Similarly, in a British study of 5958 adults, less educated adults living in the highest compared to the lowest quartile of fast-food exposure had a 2.05 (95% CI 1.08, 3.87) higher odds of being obese [35]. Although our study did not address dietary intake, our findings support this hypothesis by demonstrating that the observed environment–cardiometabolic associations are not explained by total daily step counts or MVPA.

Previous studies in this area have been smaller and/or have relied largely on self-reported measures of exposures, outcomes, and/or covariates. Our study is the first to combine laboratory-assessed measures of cardiometabolic health, GIS-derived measures of neighborhood active-living environments, and accelerometer-assessed measures of physical activity in a large population-based sample. Participants living in the most active-living-friendly neighborhoods did not accumulate more daily steps, but they did accumulate 35 min/week more MVPA, and had better BMI and SBP profiles. We found no evidence of daily step counts or MVPA mediating environment–BMI/SBP associations, suggesting that other mechanisms must explain these associations.

The strengths of our study include objective measurements of neighborhood active-living environments, markers of cardiometabolic health, and physical activity in a large sample of Canadian adults from diverse geographical settings. Two limitations should also be noted. First, our study is cross-sectional, preventing us from drawing causal conclusions. Second, we cannot exclude the possibility of residential self-selection bias resulting from healthier people choosing to live in more active-living-friendly neighborhoods, or selection bias arising from healthier people remaining in the final sample and less healthy people being excluding due to incomplete data. Third, we did not consider interaction by sex. It is possible that environment–cardiometabolic associations are different in men and women. We would encourage researchers to consider this in future studies. Lastly, it is not possible to generalize these findings to contexts with potentially very different environments and environmental health determinants. For example, factors in the environment may influence physical activity in some settings, but not in others [36].

## 5. Conclusions

Our study found that BMI and blood pressure profiles are better in adults living in neighborhoods that have more connected street networks, contain many different types of land uses, and have more people living in them. Although people who live in more active-living-friendly neighborhoods accumulate more MVPA, MVPA does not appear to explain why BMI and SBP are lower in more active-living-friendly neighborhoods. Further studies are needed to explain why active-living-friendly neighborhoods are associated with lower BMI and blood pressure.

## Figures and Tables

**Table 1:** Characteristics of the study population. Participants from the Canadian Health Measures Survey (2007–2009). MVPA, moderate-to-vigorous intensity physical activity.

**Sociodemographic Characteristics**	***n***	**Mean (SD)**
Age, years	2809	41.5 (15.1)
		**% (*n*)**
Women	2809	54.2 (1521)
Married/common-law	2807	58.3 (1635)
Education, bachelor degree or higher	2789	26.9 (751)
Ever smoker	2803	49.2 (1379)
Depressed	2805	8.5 (238)
Children <15 years in household	2809	41.0 (1151)
Working	2101	75.9 (2768)
**Cardiometabolic Measures**		**Mean (SD)**
BMI, kg/m^2^	2776	26.5 (5.4)
Systolic blood pressure, mmHg	2807	110.3 (14.7)
Hemoglobin A1c, %	2706	5.5 (0.4)
Total cholesterol/HDL cholesterol ratio	2744	4.0 (1.3)
**Physical Activity**		**Mean (SD)**
Daily step count	2251	8820 (3655)
MVPA, minutes/week	2254	167.3 (150.3)
**Neighborhood Features**		**Mean (SD)**
Active-living environment index	2809	0.06 (2.15)
Street connectivity, ≥3 way intersections/km^2^	2809	55 (31)
Land use mix, Range: 0 to 1	2809	0.22 (0.24)
Population density, population count/km^2^	2809	5381 (25,146)

**Table 2:** Mean differences in the markers of cardiometabolic health across quartiles of an index of neighborhood active-living friendliness (95% confidence intervals). Participants from the Canadian Health Measures Survey (2007–2009).

*Model* ^a,b^	Body Mass Index (kg/m^2^) ^c^	Systolic Blood Pressure (mm/Hg)	Hemoglobin A1c (%)	Total Cholesterol/HDL Cholesterol Ratio
	*n* = 2180	*n* = 2179	*n* = 2113	*n* = 2136
*Model 1*				
Quartile 2	−0.39 (−0.93 to 0.16)	−0.50 (−2.14 to 1.14)	0.01 (−0.03 to 0.05)	−0.01 (−0.16 to 0.14)
Quartile 3	−1.24 (−1.79 to −0.69)	−0.77 (−2.42 to 0.88)	−0.01 (−0.04 to 0.03)	−0.15 (−0.30 to 0.01)
Quartile 4	−1.49 (−2.03 to −0.95)	−2.82 (−4.46 to −1.18)	−0.04 (−0.07 to 0.001)	−0.17 (−0.32 to −0.02)
*Model 2*				
Quartile 2	−0.05 (−0.56 to 0.45)	0.41 (−0.99 to 1.81)	0.03 (−0.001 to 0.07)	0.08 (−0.06 to 0.21)
Quartile 3	−0.90 (−1.41 to −0.38)	−0.27 (−1.69 to 1.14)	0.01 (−0.03 to 0.04)	−0.06 (−0.20 to 0.08)
Quartile 4	−0.98 (−1.50 to −0.46)	−1.63 (−3.07 to −0.19)	−0.004 (−0.04 to 0.03)	−0.01 (−0.15 to 0.13)
*Model 3*				
Quartile 2	−0.08 (−0.59 to 0.42)	0.29 (−1.11 to 1.68)	0.04 (0.001 to 0.07)	0.08 (−0.06 to 0.21)
Quartile 3	−0.94 (−1.45 to −0.43)	−0.50 (−1.91 to 0.91)	0.01 (−0.02 to 0.05)	−0.05 (−0.19 to 0.09)
Quartile 4	−1.03 (−1.55 to −0.51)	−2.02 (−3.47 to −0.58)	0.0001 (−0.04 to 0.04)	−0.02 (−0.16 to 0.12)
*Model 4*				
Quartile 2	0.003 (−0.50 to 0.51)	0.41 (−0.99 to 1.81)	0.04 (0.001 to 0.07)	0.09 (−0.05 to 0.23)
Quartile 3	−0.81 (−1.32 to −0.29)	−0.29 (−1.71 to 1.13)	0.01 (−0.02 to 0.05)	−0.02 (−0.16 to 0.12)
Quartile 4	−0.79 (−1.31 to −0.27)	−1.65 (−3.10 to −0.20)	0.002 (−0.03 to 0.04)	0.02 (−0.12 to 0.17)

^a^ Quartile 1: Least connected/mixed/populated neighborhoods (Reference category); Quartile 4: most connected/mixed/populated neighborhoods. ^b^ Model 1: Unadjusted. Model 2: Adjusted for age, sex, married/common-law, and education. Model 3: Adjusted for age, sex, married/common-law, education, smoking status, depressed mood, having children at home aged <15 years, working status, BMI, and daily step count. Model 4: Adjusted for age, sex, married/common-law, education, smoking status, depressed mood, having children at home aged <15 years, working status, BMI, and MVPA. ^c^ Models 3 and 4 are not adjusted for BMI.

## Supplementary Materials

The following are available online at http://www.mdpi.com/1660-4601/15/10/2079/s1, Table S1: Characteristics of the study population, by those included and excluded from the present study. Participants from the Canadian Health Measures Survey (2007–2009).
