# Supplementary material for: Laboratory-Assessed Markers of Cardiometabolic Health and Associations with GIS-Based Measures of Active-Living Environments
Source: Int J Environ Res Public Health. 2018 Sep 21;15(10):2079. doi: 10.3390/ijerph15102079 (PMC6211066; doi:10.3390/ijerph15102079)
Supplement: Supplementary file 1 [file ijerph-15-02079-s001.zip › ijerph-338891-supplementary.docx]

**Supplementary Materials: Laboratory-Assessed Markers of Cardiometabolic Health and Associations with GIS-Based Measures of Active-Living Environments**

Samantha Hajna, Kaberi Dasgupta and Nancy A. Ross

**Table S1.** Characteristics of the study population, by those included and excluded from the present study. Participants from the Canadian Health Measures Survey (2007–2009).

|  | **Included**  ***n* = 2809** | | **Excluded**  ***n* = 917** | |
| --- | --- | --- | --- | --- |
| **Socio-Demographic Characteristics** | ***n*** | **Mean (SD)** | ***n*** | **Mean (SD)** |
| Age, *years* | 2809 | 41.5 **(**15.1) | 917 | 62.9 **(**11.6) |
|  |  | **% (*n*)** |  | **% (*n*)** |
| Women | 2809 | 54.2 (1521) | 917 | 50.2 (460) |
| Married/common-law | 2807 | 58.3 (1635) | 917 | 66.2 (607) |
| Education (bachelor degree or higher) | 2789 | 26.9 (751) | 911 | 17.1 (156) |
| Ever smoker | 2803 | 49.2 (1379) | 914 | 57.0 (521) |
| Depressed | 2805 | 8.5 (238) | 917 | 10.9 (100) |
| Children <15 years in household | 2809 | 41.0 (1151) | 917 | 13.3 (122) |
| Working | 2101 | 75.9 (2768) | 792 | 43.1 (341) |
| **Cardiometabolic Measures** |  | **Mean (SD)** |  | **Mean (SD)** |
| BMI, kg/m^2^ | 2776 | 26.5 (5.4) | 915 | 29.7 **(**5.8) |
| Average systolic blood pressure, mmHg | 2807 | 110.3 **(**14.7) | 917 | 122.9 **(**17.1) |
| Hemoglobin A1c, *%* | 2706 | 5.5 **(**0.4) | 878 | 6.1 **(**1.0) |
| Total cholesterol/HDL cholesterol ratio | 2744 | 4.0 **(**1.3) | 894 | 4.1 (1.2) |
| **Physical Activity Profile** |  | **Mean (SD)** |  | **Mean (SD)** |
| Steps per day | 2251 | 8820 **(**3655) | 714 | 6923 **(**3426) |
| MVPA. minutes/week | 2254 | 167.3 **(**150.3) | 714 | 94.2 (113.2) |

© 2018 by the authors; licensee MDPI, Basel, Switzerland. This article is an open access article distributed under the terms and conditions of the Creative Commons by Attribution (CC-BY) license (http://creativecommons.org/licenses/by/4.0/).
